# Data on Fourier transform-infrared of *Cosmos caudatus* Kunth. tissues analyzed with chemometric analysis

**DOI:** 10.1016/j.dib.2018.06.025

**Published:** 2018-06-19

**Authors:** Darvien Gunasekaran, Hamidun Bunawan, Ismanizan Ismail, Normah Mohd Noor

**Affiliations:** Institute of Systems Biology, Universiti Kebangsaan Malaysia, 43600 UKM Bangi, Selangor, Malaysia

## Abstract

In this dataset, we differentiate four different tissues of *Cosmos caudatus* Kunth (leaves, flowers, stem and root) obtained from UKM Bangi plot, based on Fourier transform-infrared spectroscopy. Different tissues of *C. caudatus* demonstrated the position and intensity of characteristic peaks at 4000–450 cm^−1^. Principal component analysis (PCA) shows three main groups were formed. The samples from leaves and flowers were found to be clustered together in one group, while the samples from stems and roots were clustered into two separate groups, respectively. This data provides an insight into the fingerprint identification and distribution of metabolites in the different organs of this species.

**Specifications Table**

**Table:** 

Subject area	Biology
More specific subject area	Plant Sciences
Type of data	Figure
How data was acquired	Fourier Transform-Infrared spectroscopy (Perkin-Elmer Frontier^™^ with a spectrum software version 10.3)
Data format	Analyzed
Experimental factors	Different organs of *Cosmos caudatus* as leaves, flowers, stem and root were analysed using Fourier Transform-Infrared (FTIR) spectroscopy coupled with chemometric analysis.
Experimental features	FTIR coupled with chemometric analysis was used to primarily discriminate and to identify functional groups or chemical bonds in several accessions of *Cosmos caudatus* in Peninsular Malaysia, specifically Bangi. This approach is the first fingerprint identification for this plant.
Data source location	UKM plot, Bangi, Malaysia (2.922165, 101.788304)
Data accessibility	The data is available with this article.

**Value of the data**

• Fourier transform-infrared (FTIR) is a fast, effective and non-destructive procedure to provide unique fingerprints without any sample pretreatment [1], [2], [3].
• FTIR spectroscopic data in combination with multivariate statistical analysis were performed to discriminate between tissues of *Cosmos caudatus*.
• FTIR and multivariate analysis are able to separate tissues of *Cosmos caudatus* into three main clusters.
• This provides an insight on the distribution of metabolites that are responsible for biochemical processes in different plant tissues.

## 1 Data

**Fig. 1:**
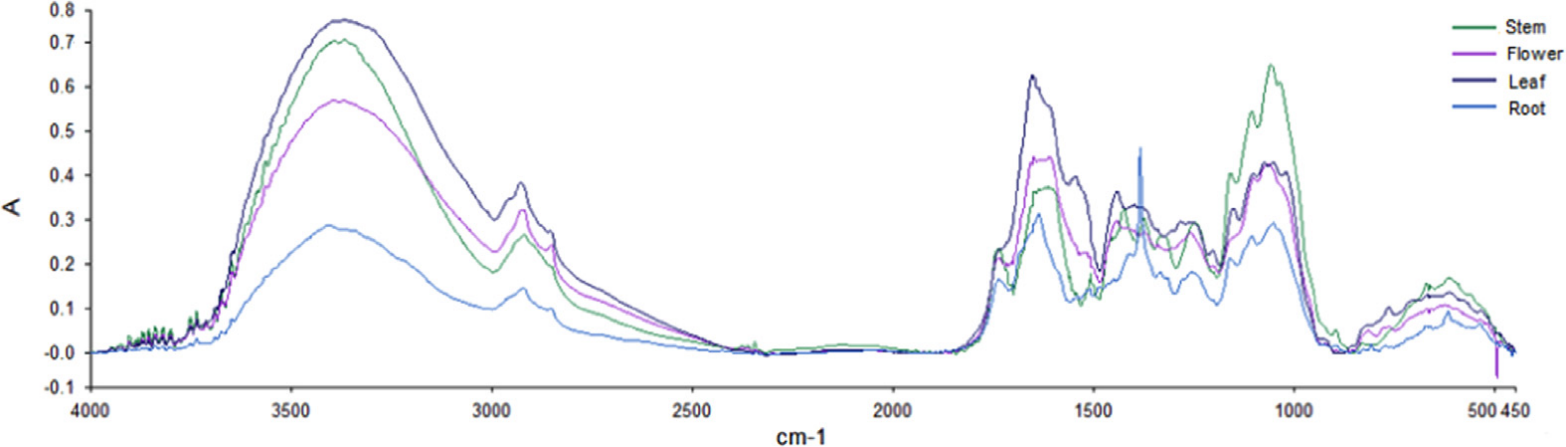
Several major functional groups in the different organs of *Cosmos caudatus* been identified from FTIR spectra (4000–450 cm^−1^).

**Fig. 2:**
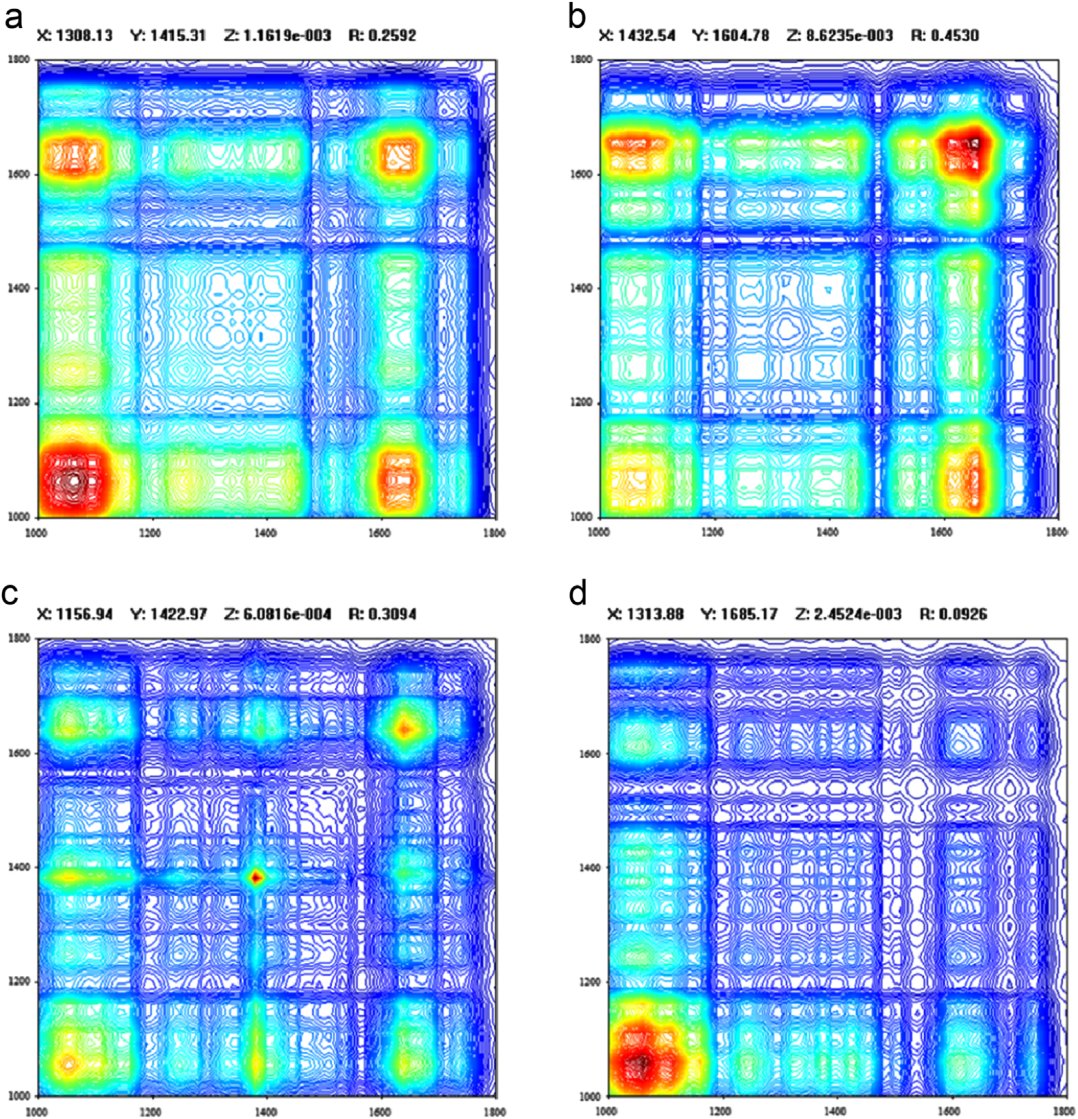
Synchronous 2D IR correlation spectra obtained using a 2D-IR correlation analysis (TD: IR 2D COS) software from different organs of *Cosmos caudatus.* (a) 2D-IR synchronous correlation spectra flower. (b) 2D-IR synchronous correlation spectra leaf. (c) 2D-IR synchronous correlation spectra root. (d) 2D-IR synchronous correlation spectra stem.

**Fig. 3:**
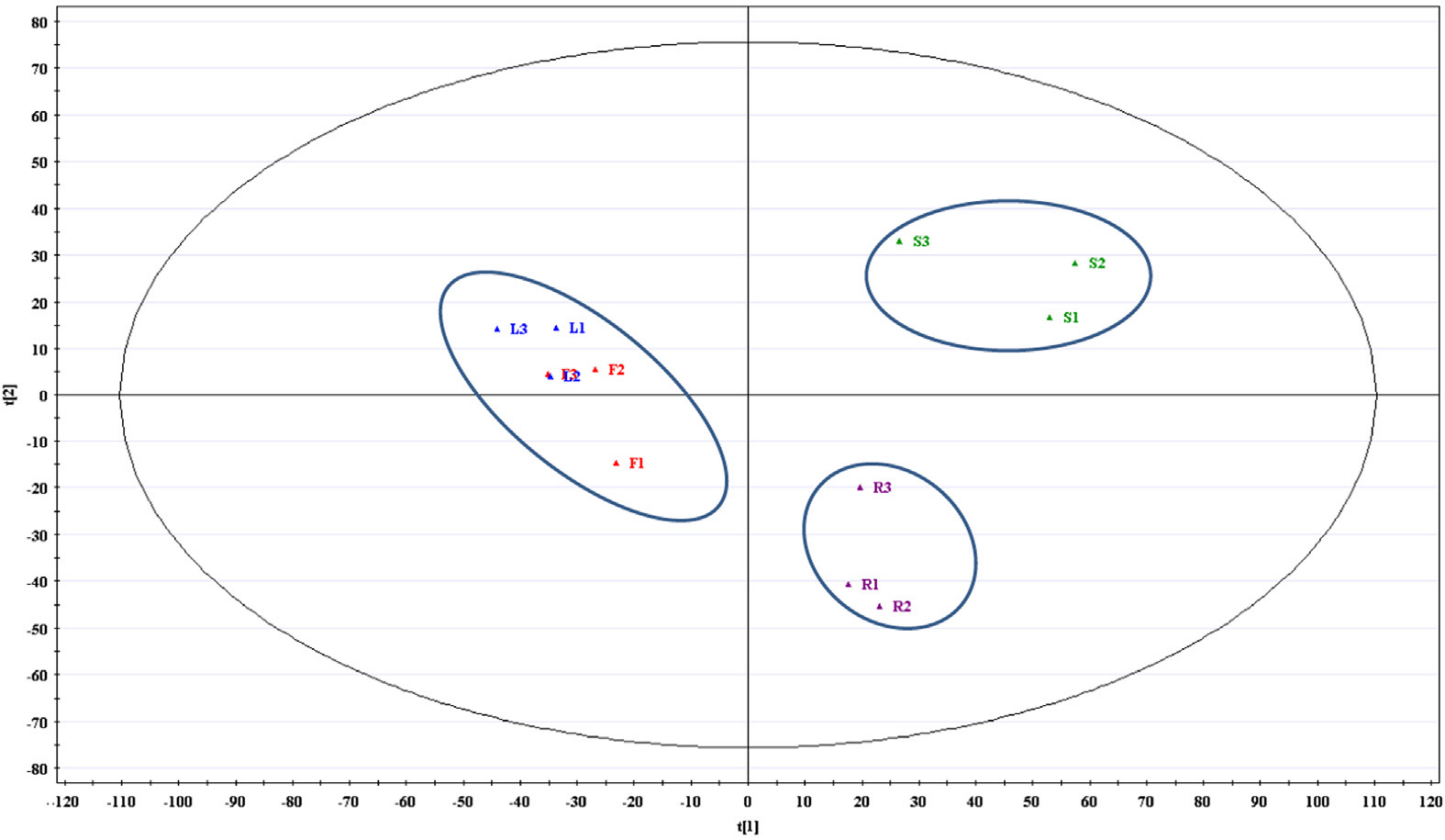
2D score scatter plot of principal component analysis (PCA) obtained from SIMCA-P+ 12 software distinguish the different organs of *Cosmos caudatus* into three clusters in terms of metabolites distribution using multivariate analysis.

**Fig. 4:**
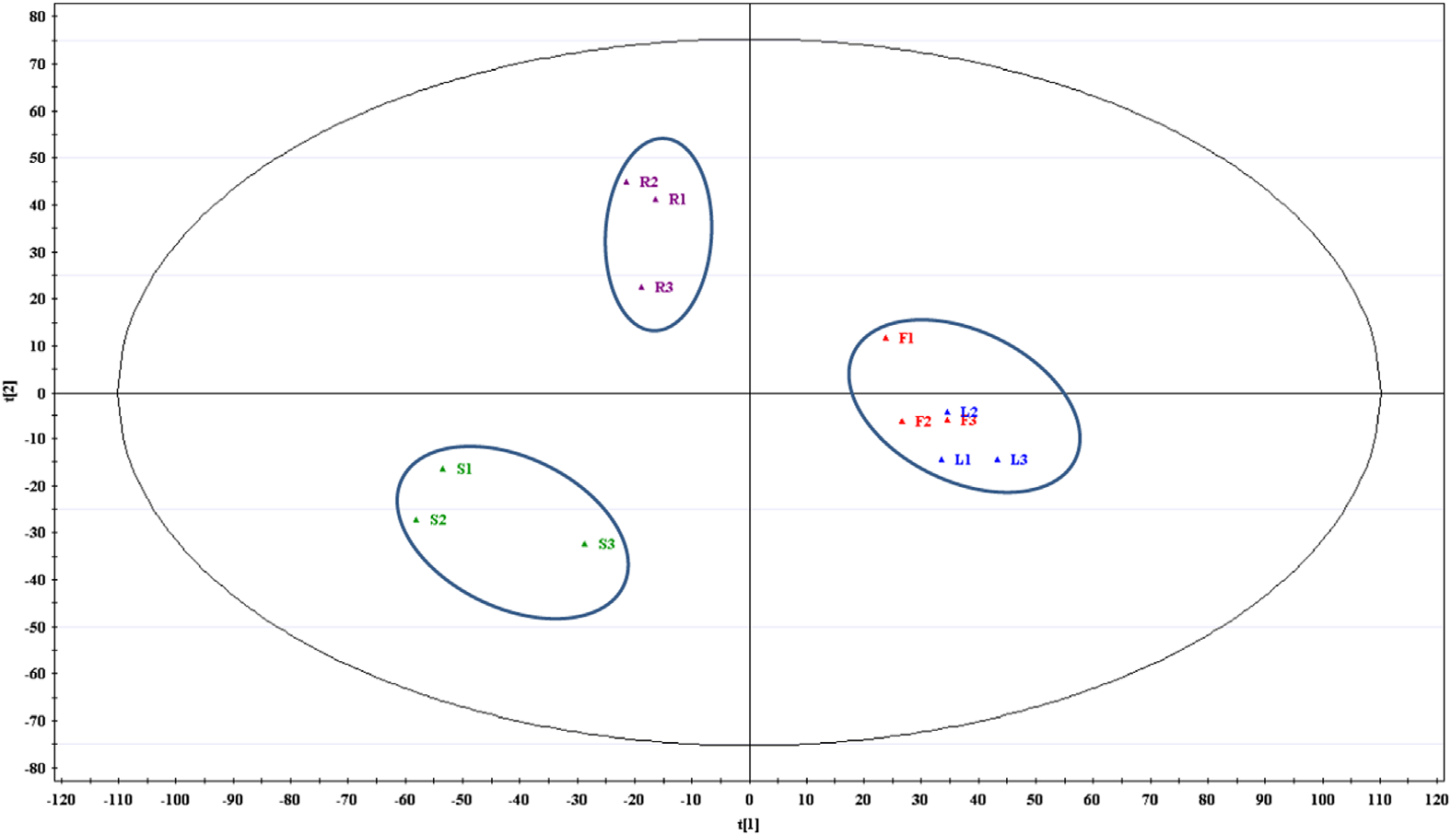
2D score scatter plot of partial least squares discriminant analysis (PLSDA) obtained from SIMCA-P+ 12 software distinguish the different organs of *Cosmos caudatus* into three clusters in terms of metabolites distribution using multivariate analysis.

FTIR spectra (4000–450 cm^−1^) identified several major functional groups in the different organs of *Cosmos caudatus* from UKM plot, Bangi, Malaysia (2.922165, 101.788304) (Fig. 1). Based on the obtained peaks from spectra, it can be seen that hyroxyl (O-H) and putative carbonyl (C-O & C=O) were abundantly present in the sample at 3500–3000, 1750–1500 and 1200–1000 cm^−1^ respectively. Principal component analysis (PCA) and partial least squares discriminant analysis (PLSDA) both revealed three major clustering groups: samples from leaves and flowers were clustered together in one group while samples from stems and roots were in another two groups respectively (Figs. 3 and 4). The synchronous 2D IR correlation spectra (Fig. 2) shows the distribution of functional groups in different organs of *Cosmos caudatus*. 2D-IR correlation spectra make complex spectra consisting of a large number of peaks easier to understand and helps in determining several interactions that occur at intra and inter molecular levels in this plant [4].

## 2 Experimental design, materials and methods

### 2.1 FTIR absorption spectra

Sample organs of *Cosmos caudatus* from UKM plot, Bangi, Malaysia were collected and the GPS location was recorded as 2.922165, 101.788304. FTIR analysis was conducted using Perkin-Elmer Frontier^™^ with spectrum software version 10.3 (Perkin-Elmer, USA) for sample discrimination. Freeze-dried samples fashioned into KBr pellet disks were placed into a holder for transmission IR spectral analysis. Spectra of mid infrared (MIR) from an accumulation of 16 scans in the range of 4000–450 cm^−1^ were recorded with resolution of 1 cm^−1^ (Fig. 1). Analysis using 2D-IR was performed correspondingly with the tablet of freeze dried specimens set into a holder with a temperature controller (Specac, USA) constantly warmed to 2 °C min^−1^ of an increasing rate of the transmission. IR spectra were gathered at temperature interims of 10 °C from 40 to 120 °C to frame dynamic FTIR spectra. 2D-IR synchronous correlation spectra were obtained from an arrangement of dynamic spectra dissected utilizing a 2D-IR correlation spectra (TD: IR 2D COS) programming created by Tsinghua University, China (Fig. 2). All the analyses were done using three biological replicates. The baselines of all the data sets were corrected.

## 3 Statistical and multivariate analysis

The areas obtained from IR spectra were normalized by sum prior to statistical and multivariate analyses. Differences between combined data of different organs were analyzed using one-way ANOVA analysis to filter the significant data in which a value of *p*<0.05 was considered to be significant. The data were inserted in Excel.csv format and the analyses were carried out using MetaboAnalyst 3.0 online software. The significant data then were uploaded in SIMCA-P+ 12.0 software and were scaled prior to differentiation and classification of the samples in graphical method of PCA (Fig. 3) and PLS-DA (Fig. 4) [5].
