# Supplementary material for: Data on Fourier transform-infrared of Cosmos caudatus Kunth. tissues analyzed with chemometric analysis
Source: Data Brief. 2018 Jun 19;19:1423–7. doi: 10.1016/j.dib.2018.06.025 (PMC6141152; doi:10.1016/j.dib.2018.06.025)
Supplement: Supplementary file 1 — Supplementary material [file mmc1.zip › Conflict of Interest.pdf]

Conflict of Interest

The authors declare there is no conflict of interest.
